# Mediation Effect of Perceived Fitness on the Relationship between Self-Efficacy and Sport Practice in Spanish Adolescents

**DOI:** 10.3390/ijerph17238800

**Published:** 2020-11-26

**Authors:** Silvia Arribas-Galarraga, Izaskun Luis-de Cos, Gurutze Luis-de Cos, Saioa Urrutia-Gutierrez

**Affiliations:** 1 Departament of Musical, Visual Arts and Physical Education Didactics, Euskal Herriko Unibertsitatea/Universidad del País Vasco, 48940 Leioa, Spain; silvia.arribas@ehu.eus (S.A.-G.); saioa.urrutia@ehu.eus (S.U.-G.); 2 Physical Activity and Sport Sciences Section, Euskal Herriko Unibertsitatea/Universidad del País Vasco, 48940 Leioa, Spain; gurutze.luis@ehu.eus

**Keywords:** self-efficacy, perceived fitness, sport practice

## Abstract

There has been a decrease in sports practices among the adolescent population, and several authors have tried to identify variables that can explain this decrease by analyzing psychosocial aspects such as perceived fitness and self-efficacy. Therefore, the purpose of this research is to examine the association of perceived fitness and self-efficacy with sport practices and to determine whether perceived fitness is a mediator of the association between self-efficacy and sport practice in Spanish adolescents. The sample was composed of 882 students between 13 and 17 years old from Gipuzkoa (Spain). A descriptive, correlational and direct/indirect effect approach was used, using the PROCESS macro for Statistical Package for the Social Sciences (SPSS). Among the results obtained, it is highlighted on the one hand, that perceived fitness significantly correlates with both self-efficacy and sport practice, on the other hand, it is confirmed that perceived fitness is a mediator in the relationship between self-efficacy and sports practice. This finding highlights the importance of psychosocial aspects in efforts to increase sports practice.

## 1. Introduction

The awareness of living a healthy life from its broadest conception is becoming increasingly important in actual society. However, the World Health Organization (WHO [1] warns that sedentary lifestyles are on the rise among the general population, and more specifically among adolescents. In terms of caring for the body, the aim should be not only to maintain a balanced diet but also to achieve a healthy body, that is, mentally and physically healthy [1], which means including sport and physical activity in one’s daily routine.

Several cross-sectional studies on the sports activity habits of the Spanish population have highlighted a decrease in sports practice among adolescents [2,3,4]. This period of life requires special attention, not only because it is the period when structural changes on a physical and psychological plane take place [5], but also because it is the time when future habits are acquired [3,4,5,6,7].

This fact has led several authors to try to identify variables that can explain this change that is occurring in the practice of sports activity among adolescents [8,9,10]. In this respect, research has highlighted both individual and contextual factors, responsible for explaining the establishment of sport attitudes and habits [11,12,13].

One of the most recognized classical theories of human behavior is Bandura’s social cognitive theory of human behavior [14], which gives great importance to self-efficacy expectations, considering them one of the most influential mechanisms in explaining behavior. In addition, Bandura [15] points out that self-efficacy is an antecedent to the action and can act as a motivator and cognitive guide of the action, being a precursor of the choice of activities, effort and persistence in the chosen activities [16].

The scientific literature shows that self-efficacy plays a relevant role in the adherence of sports practice, defining the type of activity in which adolescents are involved [17]. Various studies carried out in the field of sports and physical activity have concluded that feeling capable of achieving the goals set is a factor that increases practice [18,19,20,21,22].

Bandura [15] defined self-efficacy as an individual’s judgments about his or her abilities and, based on these judgments, they will organize and execute their actions in a way that allows them to achieve the desired performance. Self-efficacy has also been analyzed in relation to participation in physical activity, having special relevance in the establishment and maintenance of this practice [23,24,25,26]. Many scientific research studies have postulated that there is a direct relationship between self-efficacy and the practice of sports and physical activity. In the same way, it has been observed that individuals with higher levels of self-efficacy practice more sport; consider it more accessible, exciting and motivating; are more consistent in the achievement of the proposed goal; feel more satisfied; achieve, consequently, better sports results [27,28,29,30,31].

However, self-efficacy is a variable that is constantly elaborated and re-elaborated by processing and integrating information from different sources [14]. According to Bandura [15], personal achievements provide information about previous executions; observation of other’s behavior, which is used as a guide for one’s own actions; verbal persuasion, which makes it possible to use the information received from the outside; self-perception of body’s physiological state, which includes the changes in the physical form that are perceived. These are the sources of information that could lead to variations in the perception self-efficacy. Thus, the cognitive processing of the information obtained from the sources provides details on the level of self-efficacy that a person perceives when facing a certain task, which, at the same time, acts as a determinant of the sports behavior [29]. Several studies on the sources of information show, on the one hand, that the experimentation of good executions on an athletic task is effective in improving the beliefs of self-efficacy on that action [32], on the other hand, that the visualization of videos of professional soccer matches improved the effectiveness of athletes in their actions and that the reinforcement of the ego can be used to improve and maintain self-efficacy [33]. Regarding the physiological state, a study carried out by Feltz and Riessinger [34] proposes that athletes with low physical form, high level of fatigue, fear and pain, have a low level of self-efficacy.

Perceived fitness is a set of perceptions about personal physical fitness, either health or performance perspective. To assess this concept, personal perception of fitness is established as an indicator of these perceptions. These perceptions may be information that the “self” collects in order to create its physical self-concept, that is understood from the multidimensional and hierarchical perspective proposed by Fox [35], which comprises four sub-dimensions: physical ability, physical condition, physical attractiveness and strength, in which physical condition is the evaluation of confidence in personal physical fitness [36,37].

The practice of sport activity has been positively related to perceived fitness and self-efficacy [38,39,40,41], affirming that the perception of good fitness facilitates commitment to sport practice. Considering the scientific evidence, it is important to deepen our understanding as to how the variables act in this relationship.

According to the reviewed scientific literature, there is a lack in the study of the self-perception of the physiological state (perceived fitness), and more specifically in the relationship between this dimension and self-efficacy. Therefore, the aim of this study is to examine the association of perceived fitness and self-efficacy with sport practice and to determine whether perceived fitness is a mediator of the association between self-efficacy and sport practice in Spanish adolescents. We hypothesize that higher levels of perceived fitness will mediate the effect of self-efficacy on sport practice.

## 2. Materials and Methods

### 2.1. Participants

The sample under study was made up of 882 young boys and girls—48.4% of the sample were boys and 51.6% were girls resident in Gipuzkoa (Spain) and aged between 13 and 17 years (M = 14.85; S = 1.35).

### 2.2. Instrument

To measure self-efficacy, the scale extracted from the Stress-Recovery Questionnaire for Athletes (RESTQ-SPORT) by Kellmann and Kallus [42], adapted into Spanish by González-Boto, Salguero, Tuero and Márquez [43], was used. It consists of 3 items (“When I do sport, I am convinced that I can achieve what I set out to do”, “When I do sport, I am convinced that I can do it well at any time” and “When I do sport, I think that I am capable of performing any task properly”). The answers were collected, indicating the degree of agreement/disagreement, on a Likert scale from 1 to 10 where 1 is nothing and 10 is a lot. Internal consistency was 0.81 according to Cronbach’s Alpha.

To measure the perceived state of fitness, the scale extracted from the Stress-Recovery Questionnaire for Athletes (RESTQ-SPORT) by Kellmann and Kallus [42], adapted to Spanish by González-Boto et al. [43], was used. It consists of 4 items (“I feel physically strong”, “I feel full of energy”, “I am in good physical shape” and “I recover well physically”). The answers were collected, indicating the degree of agreement/disagreement, on a Likert scale from 1 to 10 where 1 is complete disagreement and 10 is complete agreement. Internal consistency was 0.86 according to Cronbach’s Alpha.

To assess sport practices, they were asked to indicate how many days per week they performed sport practices—considering sport practices to include any sport activity, organized or unorganized, that meet the conditions of a minimum duration of 60 min and a medium and vigorous intensity.

### 2.3. Procedure

To ensure representativeness of the sample, the IKERBASQUE Company has been in charge of the representativeness calculations. The sampling technique has been random and stratified in terms of age range (13–17 years) and sex (men or women). The confidence interval for the selection of the sample was 95%, with a sampling error for the total sample of about ±5%. Once this selection was made, the heads of the schools were contacted by telephone, the project was explained to them, and the relevant permits were obtained. Once an appointment had been arranged, the data were collected at the school. After a brief introduction explaining the objective of the study and clarifying any doubts, the participants proceeded to fill in the questionnaire, which took a total of 45 min. The study was carried out in accordance with the ethical standards of the Declaration of Helsinki and complies with the regulations established by the ethics committee of the University of the Basque Country (EHU/UPV) receiving a favorable report from the Ethics Committee for Research with Human Beings (ECRHB-UPV/EHU, BOPV 32, 17/2/2014, Cod.M10_2017_187).

### 2.4. Data Analysis

Firstly, descriptive statistics were calculated for all the variables under study (means and standard deviations), and the internal consistency of each factor was analyzed using Cronbach’s Alpha. In order to analyze the relationships between the variables, Pearson’s bivariate correlations were performed for the prediction model. Simple mediation analyses were performed using the macro PROCESS developed by Hayes [44] with a bootstrap threshold of 5000 and model 4. If zero was not included in the 95% confidence interval (CI) of the estimate, we concluded that the indirect effect (IE) was statistically significant. In this way, we confirmed whether the perceived fitness mediates the relationship between self-efficacy and sport practice. The standardized (β) and unstandardized (B) regression coefficients are presented for four equations: (a) the equation that regressed the mediator (perceived fitness) on the independent variable (self-efficacy), (b) the equation that regressed the dependent variable (sport practice) and the mediator, (c) the equation that regressed the independent variable and the dependent variable, and finally (d) the equation that regressed the IE of the mediator (perceived fitness) on the relationship between the independent variable (self-efficacy) and the dependent variable (sport practice).

## 3. Results

In order to obtain information about the relationship between the variables studied, bivariate correlations were carried out. The results revealed that the practice of sports and physical activity was positively and statistically significantly related to self-efficacy and perceived fitness (Table 1). A positive and statistically significant correlation was also observed between self-efficacy and perceived state of fitness.

**Figure 1:**
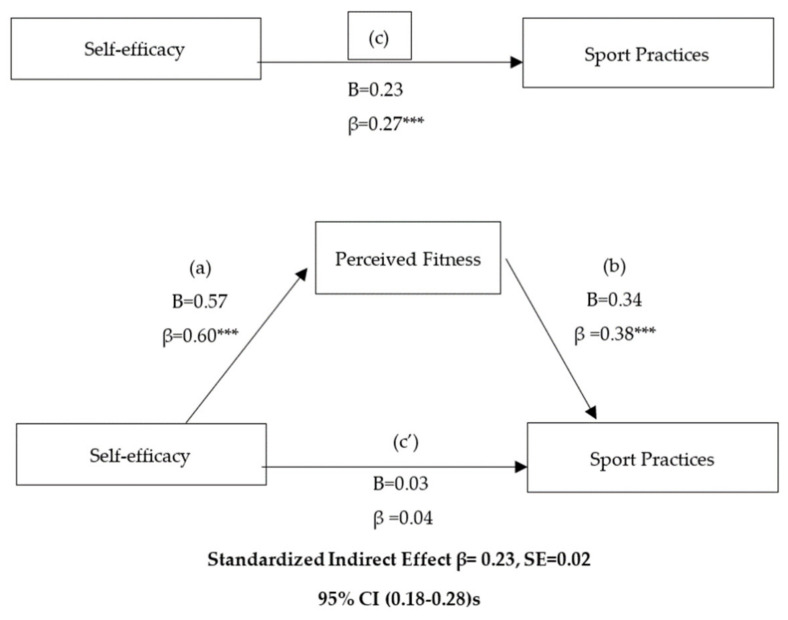
Simple mediation analysis of self-efficacy on the sport practices in relation to perceived fitness. B: unstandardized regression coefficient; β: standardized regression coefficients; SE: standard error, CI: confidence interval. *** Statistically significant at *p* = 0.000.

The results of the mediation analysis to determine whether perceived fitness acted as a mediator variable between self-efficacy (independent variable) and sport practice (dependent variable). As shown in Figure 1, the effect of self-efficacy on sport practices was mediated by perceived fitness. In the first regression equation (a), self-efficacy was positively linked to perceived fitness (β = 0.60; *p* < 0.001). In the second step (equation c), the regression coefficient of self-efficacy on sport practice was significantly associated (*p* < 0.001). In the last regression model, the mediator variable (perceived fitness) was positively associated with sport practice (equation b) (*p* < 0.001), but when perceived fitness was included in the model (equation c’), the regression coefficient turned out to be non-significant, and the relationship was removed. Finally, the indirect effect was significant (indirect effect = 0.23) (95% CI, 0.18–0.28), confirming the mediation role of perceived fitness in this model.

## 4. Discussion

The present study examined the association of self-efficacy and perceived fitness with sport practices. Our findings suggest that adolescents who perceive themselves more effective, practice more sports activities. In addition, we found that perceived fitness mediates the relationship between self-efficacy and sport practices. These results may suggest the importance of improve self-efficacy levels, and preferably increase perceived fitness levels to promote sport practices.

Increasing age is associated with a decrease in sport practices [2,4,10] and self-efficacy may be a modifiable factor that can increase levels of sport activity. The findings of the present study suggest that adolescents who consider themselves more effective present higher levels of sport participation and it is more likely that they continue practicing sport than those who consider themselves less effective [25,45,46]. The results obtained in this study support the findings of other studies [24,27,30,47], in which positive association between sport practice and self-efficacy was confirmed. This association was supported by Velazquez-Buendía et al. [48], who verified the predictive value of self-efficacy when explaining sport practice [48], reinforcing the idea of the incidence of self-efficacy in the maintenance and increase in sport practice. In this line, Bandura [15], in his social cognitive theory, postulated that the belief we have about our effectiveness is the fundamental axis to determine our actions, since it has a decisive role between the social environment and the final behavior.

Therefore, based on our results, we propose promoting actions where beliefs of self-efficacy increase, in order to ensure the continuity of adolescents’ sport practice. Taking into account different studies [3,4,5,6,7] that corroborate the idea that sport habits are established throughout adolescence, we consider that the sport habits created in this period will endure during their adulthood.

Our mediation analysis reveals that perceived fitness mediates the relationship between self-efficacy and sport practice, removing this relationship. This means that the direct effect of self-efficacy on sport practice disappears when the perceived fitness is included in the model, generating an indirect effect of self-efficacy on sport practice through the perceived fitness. The mechanisms whereby perceived fitness may influence the association between self-efficacy and sport practice are not clear. Different factors may improve self-efficacy perceptions, and the increase in physical fitness allows us to consider that an increase in the perception of physical fitness would be one of them due to its importance in adolescence. We can assume that if a change occurs in the physiological state the perception of it will modify. This idea is related to Bandura’s [15] sources of information of self-efficacy, where personal fitness provides information for the establishment of the self-efficacy.

In addition, several studies suggest that the evaluation of physical fitness explains better the benefits associated with sport practice [49,50]. Likewise, the improvement in the perceptions of fitness that adolescents experience makes them perceive fewer barriers to practicing sports [39,51,52,53]. In turn, the scientific literature affirms that the perceived state of form facilitates commitment to sports practice [40,41].

There are some limitations to our investigation. First, the cross-sectional nature of the study does not allow us to affirm that this association is derived from a relation of causality. Second, the use of a self-report measurement to assess sport practice may have elicited some degree of recall and desirability bias.

## 5. Conclusions

The present findings suggest that having higher levels of self-efficacy is associated with higher level of sport practice. Additionally, our mediation analysis reveals that perceived fitness mediates the relationship between self-efficacy and sport practice, removing this relationship.

## Figures and Tables

**Table 1:** Descriptive and correlational analyses between sports practice, self-efficacy and perceived state of fitness.

			Correlations	
	M	SD	1	2
1. Sports practice	2.7	1.01	-	-
2. Self-efficacy	6.77	1.77	0.26 **	-
3. Perceived fitness	6.84	1.71	0.38 **	0.59 **

Note: ** *p* < 0.001; M = mean; SD = standard deviation.
